# Interspecific Nematode Signals Regulate Dispersal Behavior

**DOI:** 10.1371/journal.pone.0038735

**Published:** 2012-06-06

**Authors:** Fatma Kaplan, Hans T. Alborn, Stephan H. von Reuss, Ramadan Ajredini, Jared G. Ali, Faruk Akyazi, Lukasz L. Stelinski, Arthur S. Edison, Frank C. Schroeder, Peter E. Teal

**Affiliations:** 1 Center for Medical, Agricultural and Veterinary Entomology, Agricultural Research Service, United States Department of Agriculture (USDA-ARS), Gainesville, Florida, United States of America; 2 Boyce Thompson Institute and Department of Chemistry and Chemical Biology, Cornell University, Ithaca, New York, United States of America; 3 Department of Biochemistry and Molecular Biology, and National High Magnetic Field Laboratory, University of Florida, Gainesville, Florida, United States of America; 4 Entomology and Nematology Department, Citrus Research and Education Center University of Florida, Lake Alfred, Florida, United States of America; 5 Entomology and Nematology Department, University of Florida, Gainesville, Florida, United States of America; 6 Department of Plant Protection, Ordu Universitesi, Ordu, Turkey; Instituto Butantan, Brazil

## Abstract

**Background:**

Dispersal is an important nematode behavior. Upon crowding or food depletion, the free living bacteriovorus nematode *Caenorhabditis elegans* produces stress resistant dispersal larvae, called dauer, which are analogous to second stage juveniles (J2) of plant parasitic *Meloidogyne spp*. and infective juveniles (IJ)s of entomopathogenic nematodes (EPN), e.g., *Steinernema feltiae*. Regulation of dispersal behavior has not been thoroughly investigated for *C. elegans* or any other nematode species. Based on the fact that ascarosides regulate entry in dauer stage as well as multiple behaviors in *C. elegans* adults including mating, avoidance and aggregation, we hypothesized that ascarosides might also be involved in regulation of dispersal behavior in *C. elegans* and for other nematodes such as IJ of phylogenetically related EPNs.

**Methodology/Principal Findings:**

Liquid chromatography-mass spectrometry analysis of *C. elegans* dauer conditioned media, which shows strong dispersing activity, revealed four known ascarosides (ascr#2, ascr#3, ascr#8, icas#9). A synthetic blend of these ascarosides at physiologically relevant concentrations dispersed *C. elegans* dauer in the presence of food and also caused dispersion of IJs of *S. feltiae* and J2s of plant parasitic *Meloidogyne spp.* Assay guided fractionation revealed structural analogs as major active components of the *S. feltiae* (ascr#9) and *C. elegans* (ascr#2) dispersal blends. Further analysis revealed ascr#9 in all *Steinernema* spp. and *Heterorhabditis* spp. infected insect host cadavers.

**Conclusions/Significance:**

Ascaroside blends represent evolutionarily conserved, fundamentally important communication systems for nematodes from diverse habitats, and thus may provide sustainable means for control of parasitic nematodes.

## Introduction

**Figure 1:**
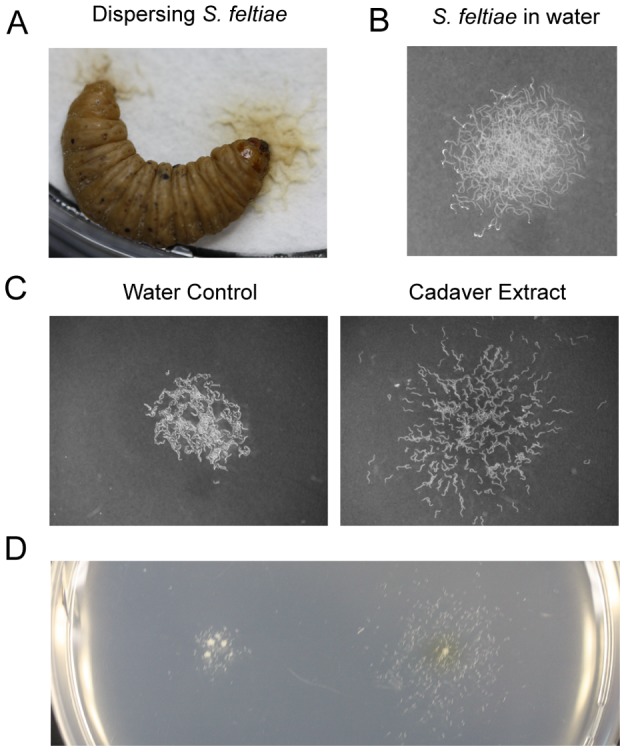
Dispersal assay. (A) Naturally dispersing infective juveniles (IJ) of *S. feltiae* from an insect cadaver. (B) Approximately 300 IJs were placed on an agar plate in water. (C) IJs were treated with either water (control) or insect cadaver extract. Images are representative of six experiments for each treatment. (D) Dispersal assay on the same plate. The illustration represents two experiments. Behavior is temperature and season dependent. Assays were conducted at RT (22±0.5°C) or under temperature controlled conditions.

Entomopathogenic nematodes (EPN), such as *Heterorhabditis* spp. and *Steinernema* spp. are obligate insect parasites that kill and consume their hosts with the aid of symbiotic bacteria [1]. *C. elegans* is typically found in decomposing plant material [2], [3]. It completes its life cycle within 3.5 days under standard laboratory conditions. When the conditions are not favorable for normal growth and development, such as high temperature, high density, or low food availability, it forms dauer larvae (alternative 3rd larvae). This specialized life stage, which does not feed and is resistant to stressful conditions [4], [5], [6], is analogous to IJs of entomopathogenic nematodes and second stage juveniles (J2) of plant parasitic root-knot nematodes [7]. (*Meloidogyne* spp) that are equally adapted to survive conditions unfavorable for normal growth and development. Upon depletion of food resources or when overcrowded, infectious juveniles (IJs) of entomopathogenic nematodes and dauer larvae of the free living nematode *Caenorhabditis elegans* display a dispersal behavior [4], [8]. This is demonstrated by highly mobile EPNs, *Steinernema feltiae* IJs, leaving a host cadaver (Fig 1A and Movie S1).

**Figure 2:**
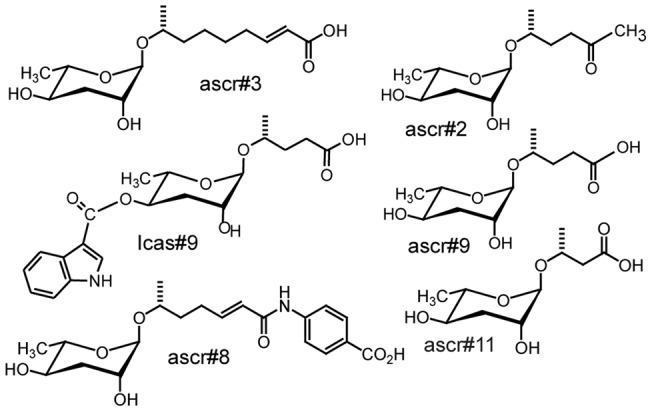
Structures of ascarosides. Structures of published ascarosides.

For *C. elegans*, entry of early larval stages (L1/ early L2) into the dauer stage is regulated by dauer pheromone [9], [10], [11], [12], [13], [14], [15], [16], which consists of several related ascarosides, derivatives of the unusual dideoxysugar ascarylose (Fig 2). Specific blends of ascarosides regulate many social behaviors in adult *C. elegans* [12], [17], [18]. For example, a mating pheromone ascaroside blend attracts adult males [12], but does not affect adult hermaphrodites. Ascr#5 with ascr#2 or ascr#3 also appear to affect aggregation of adult *C. elegans* [17]. The production and release of the *C. elegans* ascarosides change with development and environmental conditions, which is consistent with multiple functions of these signaling compounds [19].

Dispersal behavior, despite its central role in the ecology of both disease-relevant nematodes and the model organism *C. elegans* has not been thoroughly investigated for any nematode species. However, for *C. elegans* regulation of social behaviors by ascaroside pheromones strongly suggests that ascarosides might also regulate its dispersal behavior. *Caenorhabditis* and entomopathogenic nematodes (*Steinernema* and *Heterorhabditis*) are phylogenetically related bacteriovores. Both *Caenorhabditis* spp. and EPNs may utilize similar mechanisms for regulating dispersal from crowded and resource-deprived areas, including the use of a common family of signaling molecules, for example ascarosides.

## Results

### Dispersal assay

For EPN, insect cadavers are known to promote dispersal behavior of the IJ stage [20]. An assay was therefore developed to identify compounds in consumed insect cadavers that promote dispersal. The dispersal assay focused on population behavior rather than response of individual IJs. Dispersal was characterized as a group of IJs displaying increased activity and clear directional movement away from the treatment site even when the nematodes were no longer in contact with a test solution. This is different from avoidance which can be characterized as one or more nematodes moving away from a treatment site when encountering it but otherwise exhibiting a random movement behavior outside that area. Approximately 300 IJs of *S. feltiae* in 10 µl of water were placed on an agar plate (Fig 1B). Then, either 2 µl water (Fig 1C) or an aqueous extract of *Galleria mellonella* larval cadavers infected with *S. feltiae* (Fig 1D) was placed into the nematode suspension. After absorption of water into the media, nematodes were able to move freely. The water-treated nematodes remained near the site of deployment with no dispersal behavior (Movie S2). In contrast, cadaver extract-treated nematodes were very active and moved away from the point of release (Movie S3). This was the same distinct dispersal behavior observed for nematodes leaving a cadaver. Liquid chromatography-mass spectrometry (LC-MS) analyses of the cadaver extract revealed the presence of an ascaroside (ascr#9) [15] supporting the hypothesis that *S. feltiae* might utilize ascarosides as dispersal signals.

### *C. elegans* dispersal is regulated by a blend of ascarosides

**Figure 3:**
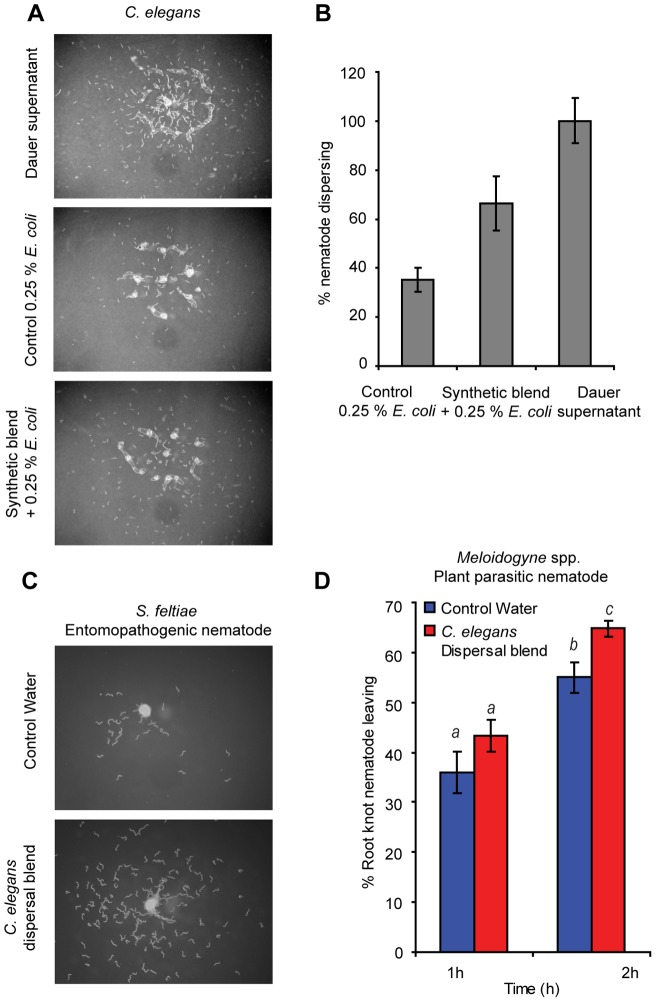
An ascaroside blend regulates *C. elegans* dispersal behavior, and the dispersal blend is recognized by other nematodes. (A) Identification of the dispersal blend. Images (∼250 nematodes) are representative of 9, 10, and 11 experiments of control (0.25% *E. coli* (HB101)), synthetic blend with 0.25% *E. coli* (HB101) and dauer supernatant, respectively. (B) Quantification using Image J (http://rsbweb.nih.gov/ij/download.html). Control vs synthetic blend student's t-test unpaired (p<0.02). (C) *S. feltiae* IJs (∼250) response to the dispersal blend; four experiments for each treatment. (D) Response of root-knot J2s (*Meloidogyne* spp., mixture of *M. incognita*, *M. javanica*, and *M. floridensis*) to the *C. elegans* dispersal blend. The data represent 19 and 20 experiments from control water and *C. elegans* dispersal blend, respectively. At 2 h, using a student's t-test, unpaired, p<0.007.

Next we investigated whether this bioassay could also be used to test for dispersal of *C. elegans* dauer larvae. For this assay, we used a growth medium from a developmentally synchronized *C. elegans* liquid culture that had produced 60% dauer larvae [19]. Following removal of all nematodes, this dauer inducing medium containing L1 and L2D secretions strongly induced dispersal behavior in the *C. elegans* dauer. Using LC-MS, the dauer forming medium was analyzed for the known ascarosides (ascr#1, ascr#2, ascr#3, ascr#4, ascr#5, ascr#6, ascr#7, ascr#8, and icas#9) and was found to contain four known ascarosides (ascr#2, ascr#3, ascr#8, and icas#9) [10], [14], [15] (Fig 2 and Fig S1A), all previously shown to individually promote dauer entry in *C. elegans* [10], [13], [14]. The concentrations of the ascarosides in the dauer forming culture medium were estimated as: ascr#2 (3.68 pmol/µl), ascr#3 (0.165 pmol/µl), ascr#8 (0.25 pmol/µl) and icas#9 (0.005 pmol/µl). A synthetic blend of these ascarosides was then tested for dispersal activity, using the dauer conditioned medium as a positive control. The media was estimated to contain approximately half of the original 0.5% *E. coli* (HB101) food source, thus 0.25% *E. coli* was added to the synthetic test samples, as well as to a water control to prevent food searching behavior induced by the absence of food. The number of dispersing nematodes was normalized to the percent of the positive control response. In the presence of just the food (negative control), approximately 35% of the dauer larvae left the release location. However, with the addition of the synthetic ascaroside blend, nearly twice as many nematodes (62%) moved away from the release location (Fig 3A and B and Fig S1B). Tested individually at physiological concentration, ascr#8 (50%) and ascr#2 (40%) gave the strongest response but all four were less active than the blend (Fig S1C). This suggested that *C. elegans* dauer larvae were able to perceive and respond to single components of the dispersal blend but the complete four-component blend was necessary to restore the activity.

### EPNs and plant parasitic nematodes sense the *C. elegans* dispersal blend

We hypothesized that many nematode species might be able to sense and respond to signals released by other nematode species. Thus ascaroside released by *C. elegans*, could function as valid avoidance signals. In our dispersal assay IJs of *S. feltiae* exhibited no noticeable movement when exposed to water, but were very active and moved away from the release location when exposed to the *C. elegans* dispersal blend (Fig 3C and Fig S1D). The mobile J2 form of wild root knot nematodes (a mixture of *Meloidogyne* spp; *M. javanica, M. floridensis,* and *M. incognita*) isolated from tomato roots were also tested for response to the *C. elegans* dispersal blend (Fig 3D). Again, more nematodes (10–12%) left the area where the *C. elegans* blend was introduced as compared to the control. Interestingly, in preliminary experiments the root knot nematodes showed no detectable behavioral response, for example in the form of increased activity, to any of ascr#9 (ascr#2 structural analog), ascr#3 and ascr#8 when tested individually at 25 ng/µl (data not shown). This suggests that at least some nematode species can respond to dispersal blends of distantly related nematode species and also that blends might be more important than individual ascarosides.

### The major components of *S. feltiae* and *C. elegans* blends are structural analogs

**Figure 4:**
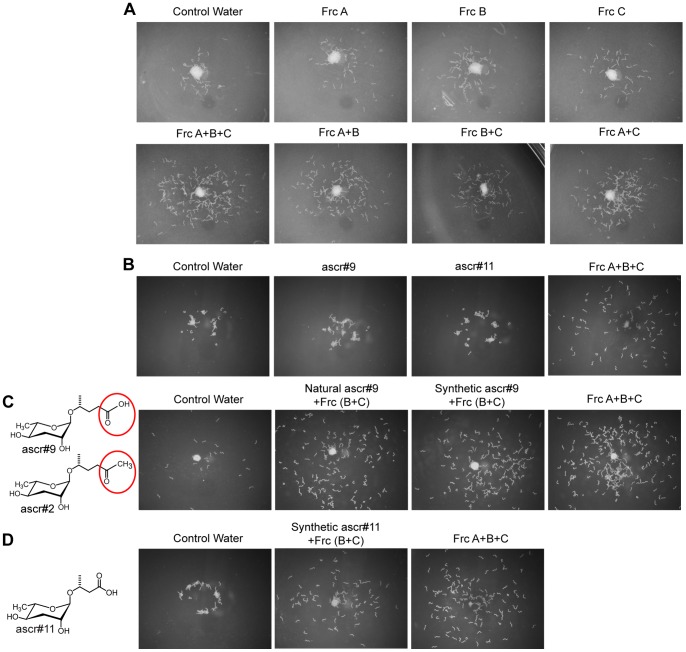
Activity guided fractionation of the *S. feltiae* dispersal pheromone. (A) Reverse phase (C18) chromatography of insect cadaver extract. The image represents two independent experiments. The estimated physiologically relevant concentration was used in the assays; Frc, fraction. (B) Testing physiologically relevant concentration of ascarosides found in Frc A (ascr#9, 40.3 pmol/µl and ascr#11, 1.3 pmol/µl). Image represents three experiments. (C) Ascr#2 and ascr#9 are structural analogs. Natural and synthetic ascr#9 were tested in combination with fraction B and C. Image represents four experiments. (D) Structure of ascr#11. It (1.3 pmol/µl) is also sufficient to cause dispersal in combination with fractions B and C. Image represents three experiments.

For characterization of the *S. feltiae* dispersal pheromone, insect host cadavers were extracted with 70% EtOH, fractionated by reverse phase (C18) chromatography and assayed (Fig 4A, B, C, and D). The bioassay revealed that a combination of all three fractions (A, B, and C) was necessary for activity (Fig 4A). Analysis of the fractions by LC-MS (Fig S2) revealed 2 ascarosides in fraction A, which were identified as ascr#9 and ascr#11 with the help of synthetic standard [15], [21]. Bioassays showed that ascr#9 and ascr#11 did not have activity by themselves at physiologically relevant concentrations (Fig 4B). This was expected because the natural fraction A was inactive when tested alone (Fig 4A). However, combining synthetic asc#9 at the biologically relevant concentration (40 pmol/µl) with natural fractions B and C, restored the original dispersal activity (Fig 4C), confirming ascr#9 as an active component of the *S. feltiae* dispersal blend. The second ascaroside found in fraction A, asc#11 also restored the full activity when combined with fraction B and C (Fig 4D), indicating that either, ascr#9 or ascr#11, by themselves are sufficient to reconstitute full dispersal activity in combination with fractions B and C.

We have previously established a developmental profile [19] for the principle *C. elegans* dispersal pheromone component, ascr#2. The primary ascaroside of fraction A in the *S. feltiae* dispersal blend, ascr#9 is a structural analog to ascr#2 (Fig 4C). Therefore, the accumulation of ascr#9, in *S. feltiae* infected insect cadavers, were analyzed in a time course experiment (Fig S3). The results showed that ascr#9 had a very similar accumulation profile as previously established for ascr#2 in *C. elegans*. Furthermore, the accumulation of ascr#9 was highest directly prior to IJ dispersal and remained constant for at least 6 days in the IJ-depleted cadaver. This was very similar to observation of ascr#2 and dauer formation for *C. elegans* [19]. In contrast, the ascr#11 profile (Fig. S3) differed from that of asc#9; it initially became detectable but not quantifiable at day 8, (dispersal day) and could not be quantified in the insect host cadaver until 14 days, 6 days after dispersal.

We also tested whether ascr#9 could substitute for its structural analog ascr#2 in the *C. elegans* dispersal blend and found this to be the case (Fig S4). Thus a cross species perception, as found with *S. feltiae*, might also be true for *C. elegans.*

### Ascr#9 could be a common component in dispersal blends of phylogenetically related EPNs

In beetles and flies, phylogenetically related species share components in their aggregation pheromone blends [22], [23]. To further test to what degree dispersal blend components were shared by phylogenetically related nematode species, insect host cadavers infected with *Steinernema* spp. and *Heterorhabditis* spp. were analyzed for presence of ascr#9, ascr#2 and ascr#11 (Fig S5). The insect host cadavers for both species were found to contain ascr#9 (Fig S5), which suggests that ascr#9 may be used by a broad range of EPN species as part of their dispersal blends. We found ascr#11 in all tested *Steinernema* spp., but not in *Heterorhabditis* spp, suggesting that ascr#11 might be specific for *Steinernema* spp. Further research will reveal to what degree *Steinernema* spp. and *Heterorhabditis* spp. nematodes utilize species-specific blends of the same components or species-specific components in their dispersal blends and to what degree they can detect and respond to these different ascaroside blends.

## Discussion

For *C. elegans*, shared and unique compositions of ascarosides regulate different behaviors. For example, the mating and dispersal blends share ascr#2, ascr#3 and ascr#8 [12], [14], [19]. The *C. elegans* mating blend is characterized by a unique component, ascr#4 [12], and the dispersal blend has a unique component, icas#9 [15]. We propose that this might be common for many nematode species. It is not yet clear if *Meloidogyne* spp. also utilize dispersal blends, but we have shown that these nematode species can detect and respond to signals released by other nematode species that are indicative of decomposing and decaying plant material. The *C. elegans* dispersal blend shows similar activity in developmentally analogous stages of phylogenetically related species, suggesting that it can be used to identify genetic targets as well as to formulate dispersal blends for control of species parasitic to plants, humans and livestock. This investigation demonstrates that several nematode species utilizes species-specific small molecule signals to regulate dispersal behavior but also that nematode dispersal behavior may be broadly induced by interspecies communication.

## Materials and Methods

### Dauer production

*C. elegans* were cultured under dauer inducing liquid culture conditions [19] in S-complete with 20,000 worms/ml and 0.5% (wet weight) *E. coli* (HB101). Nematodes were incubated at 22°C in a shaker (250 rpm) for 112 h after feeding L1 larvae nematodes. Thereafter, nematodes were treated with 1% SDS for 15 min and surviving nematodes were allowed to separate from dead nematodes on an agar plate prior to collection. After removal of the dead nematodes by vacuum, dauer animals were collected using M9 buffer and placed at 4°C.

### Rearing of *S. feltiae*

*S. feltiae* was ordered from ARBICO Organics (Tucson, AZ). *G. mellonella* larvae (Wax worms, Grubco, Hamilton, OH) were infected with a 50 *S. feltiae* dauer juveniles per larvae. After two days, the infected larvae were placed into new 6 cm diameter petri dishes and the white trap method was used to collect IJs [24].

*S. feltiae* IJs were verified by PCR using species specific primers from the ITS rDNA region as described Campos-Herrera et al [25]. PCR amplifications were performed in an MJ Research PTC 200 Peltier Thermal Cycler. Amplifications were conducted as described by Campos-Herrera et al [25] in a 25 µL final volume containing 1 µL DNA template using sterile de–ionized water and DNA prepared from *Steinernema riobrave* as negative controls. Cycling parameters were 94°C for 15 min followed by 35 cycles of denaturation at 94°C for 30 s, annealing at 59°C for 20 s and extension at 72°C for 20 s with a final extension of 72°C for 10 min. Amplicon sizes were verified through electrophoresis on a TAE 2% agarose gel and visualized in the UVP BioDoc–itTM System.

The *S. feltiae* primer produced specific amplification for all samples containing IJs from lab populations in conventional PCR. The primers showed no amplification for *S. riobrave* and de-ionized water controls.

### Rearing of root knot nematodes

Infected tomato plants were collected from field sites in Florida. Roots were inspected for root knot infection and root knot nematode eggs were collected as described by Hussey and Barker [26] with modifications. The infected roots were treated with 1% bleach for 2 min. Eggs released from egg mass matrices were collected with a nested filter system (85 µm) to collect plant debris and 25 µm nylon filters (Nytex) to collect eggs. Eggs were washed thoroughly with MILLI-Q water and placed onto a 20 µm filter on an 8 cm diameter petri dish with a small amount of water to hatch at RT for 3 days.

Root-knot nematodes extracted from infested tomato roots were identified based on morphology and isozyme phenotyping for esterase (EST) and malate dehydrogenase (MDH). Morphological identifications were done using perineal patterns of mature females as described by Eisenback [27]. Briefly, isozyme phenotyping was done using 25 young egg-laying females. Extracts of females dissected directly from root system were run on two polyacrylamide gel electrophoresis (PAGE) [28]. One gel was stained for both MDH and EST activity [29], whereas a second was stained only for EST.

### Identification of *C. elegans* dispersal blend

Liquid cultures that induced 60% dauer (2 experiments) and 40% dauer after 67 h of feeding L1s were analyzed using LC-MS. Four ascarosides were common to all three liquid media. The concentrations of each were measured from the liquid cultures that produced 60% dauers.

### Dispersal assay

*S. feltiae* IJs were washed with MILLI-Q water three times and incubated in 6 cm petri dishes for 36 h with a small amount (4–5 ml) of MILLI-Q water. The following day, nematodes were placed on a 10.7 g/L agar with gel strength 1010 g/cm^2^ (PhytoTechnology Lab. Shawnee Mission, KS). Nematode behavior was assayed on multiple plates with internal plate replicates to rule out the possibility that behavior was affected by plate composition. Approximately 300 IJs in 10 µl water were placed on an agar medium and the test compounds or extracts were placed into 1–2 µl to the nematode suspension. Upon absorption (∼15 min) of the liquid, the freely moving nematodes were video-recorded for 5–10 min. Dispersal behavior is temperature and season dependent. During winter, the assay is effective at RT (22±1°C). During summer, the assay requires a temperature-controlled environment due to effects on nematode behavior above 23°C.

*C. elegans* dauer juveniles were washed with MILLI-Q water 3 times and placed into 6 cm petri dishes with a small amount of water and rested overnight. Approximately 200–300 nematodes in 10 µl of water were placed on an agar plate and 2 µl of treatment was added. The liquid culture that produced 60% dauer animals was centrifuged and filtered with a 0.45 µm filter and used as a positive control for dispersal. Thereafter, media were lyophilized and resuspended in MILLI-Q water 5 times and 2 µl to 10 µl of nematode suspension was used for assay. As a negative control, 0.5% *E. coli* (HB101) was prepared in S-complete, lyophilized and adjusted to the final volume of 0.25% *E. coli* in the assay. The dispersal behavior was observed for 12–15 min.

Dispersal assay of root knot nematode: The root knot nematodes that hatched within 1–3 days were collected and washed with MILLI-Q water 3 times using 10 µm nylon filters (Nytex). Thereafter, they were placed into a 1.5 ml Eppendorf tube. The nematodes in 10 µl water were placed on an agar plate. Nematodes found at locations away from where they were originally placed were counted at 1 and 2 h. Each treatment was normalized using the total number of nematodes that were deployed. For each treatment, 20 experiments were conducted on three different days. The nematode density was ∼30 per plate in 14 experiments and 100 per plate in 6 experiments. The experiments were conducted in the morning.

### Quantifying of *C. elegans* dispersal

Nematodes were quantified using Image J software (http://rsbweb.nih.gov/ij/download.html). The number of nematodes visualized and counted using Image J is illustrated in Fig S1B. Nematodes inside the circle were subtracted manually from the total number of worms counted because of their location of placement. Each treatment was replicated 9–11 times. The graph is normalized to the positive control, the dauer conditioned medium.

### Purification of the *S. feltiae* dispersal blend

Activity guided fractionation was conducted as described by Srinivasan et al [12] with modifications. A total of 33 insect host cadavers (G. *mellonella* larvae) were placed into 70% EtOH and stored at −20°C until extraction. The insect cadavers were homogenized using 1 g of ceramic zirconium beads (1.25 mm) (ZIRMIL) in 2 ml tubes for 37 sec using a Precellys24 (http://www.precellys.com) homogenizer. Samples were centrifuged for 15 min at 18400 rcf and the supernatant was lyophilized and resuspended in MILLI-Q water. The dispersal activity of nematodes was tested using the dispersal assay described above and a physiologically relevant concentration of insect host cadaver extract or fractionated extract. To facilitate calculations for physiologically relevant concentration of the ascarosides, wax worm volume was estimated at ∼200 µl; the average weight of wax worms was 232+57 mg (n = 19).

The first reverse-phase solid-phase extraction was performed using Sep-Pak Plus C18 cartridges (Waters corporation, Milford, MA). The initially collected flow through was termed Fraction A. Thereafter, the column was washed with water, collected and saved. Subsequently, the column was eluted with 50% (Fraction B) and 90% MeOH (Fraction C). The fractions were tested for dispersal activity both individually and in combination. Also individual fractions were analyzed by LC-MS. Fraction A contained ascr#9, which was collected by LC-MS and tested for activity with Fraction B+C.

### Time course study of *S. feltiae* ascaroside production

The one-on-one assay method [24] was used. One wax worm was placed in one well of a 24 well plate. For infection, 50 *S. feltiae* IJ were placed in 25 µl of water in each well. After 24 h, the plates were examined for infected insect larvae and those infected were placed in a white trap [24], and those that were infected after 48 h were placed on a separate white trap. Initial sampling was made after 48 h. Thereafter, samples were taken every day for 9 days and once at day 14. For each experiment, samples were comprised of six insect host cadavers. The insect cadavers were placed into a 2 ml Eppendorf tube with 1 ml of water. The cadavers were punctured with a needle to allow dissipation of internal contents and then vortexed. Samples were centrifuged at 3380 rcf for 10 min and 0.5–1 ml of the supernatant was recovered. The supernatant was frozen at −20°C and then lyophilized. Thereafter, it was resuspended in 200 µl of 50% MeOH and diluted 1∶1 with 0.1% formic acid. Sample pH was 4.3. From that, 20 µl of sample was injected to LC-MS for analysis of ascr#9.

### Asrc#9 in insect host cadavers of *Steinernema* spp and *Heterorhabditis* spp

Insect hosts (*G. mellonella*) were infected with *H. bacteriophora, H. zealandica, H. floridensis, S. carpocapsae, S. riobrave,* or *S. diaprepesi*. When nematodes began to emerge from insect cadavers, they were placed into 1.5 ml of 70% EtOH and stored at −20°C until use. Thereafter, insect cadavers were homogenized using 1 g of ceramic zirconium beads (1.25 mm) (ZIRMIL) in 2 ml tubes for 39 sec using a Precellys24 (http://precellys.com) homogenizer. The homogenized cadavers were centrifuged at 3380 rcf for 10 min. The supernatant was diluted with 1 ml of HPLC water and placed at −20°C and then placed into a speed vac (Speed Vac Plus SC210A, Savant) overnight. Each cadaver extract was re-suspended in 1 ml of 50% MeOH and centrifuged at 18400 rcf for 15–20 min. Thereafter, samples were diluted in a 1∶1 ratio with 0.1% formic acid, yielding sample pH of 4.2. Presence or absence of arc#9 was determined by LC-MS.

### LC-MS analysis

The method used for the ascaroside analysis was described by Kaplan et al [19].

## Supporting Information

Movie S1 **Natural dispersion of entomopathogenic nematodes** (***S. feltiae***) **from insect** (***G. mellonella***) **host cadaver.**
(MP4)

Movie S2 **Dispersal assay:** ***S. feltiae*** **IJs treated with water.** The nematodes are calm with slow movement in random directions.
(MP4)

Movie S3 **Dispersal assay:** ***S. feltiae*** **IJs treated with insect cadaver extract are active and disperse away from center.**
(MP4)

Figure S1 ***C. elegans*** **dispersal blend, quantification and response of** ***S. feltiae*** **to** ***C. elegans*** **dispersal blend.** (A), Structures of the ascarosides for the dispersal blend. (B), Quantification of dispersing *C. elegans* using Image J. Second column shows inverted pictures and the third column shows the counted nematodes. (C), Contribution of individual ascarosides to the activity of the synthetic blend. Ascr#2, (3.68 pmol/µl), ascr#3 (0.165 pmol/µl), ascr#8 (0.25 pmol/µl), and icas#9 (0.005 pmol/µl). Seven experiments were done for each treatment. +, present and −, absent. Student's t-test, unpaired (p<0.05). (D), *S. feltiae* response to *C. elegans* dispersal blend visualized within an entire plate.
(TIF)

Figure S2 **LC-MS ion chromatograms of fraction A.** First panel shows ascr#11 at m/z 279, second panel shows ascr#9 at m/z 293.
(TIF)

Figure S3 **Ascr#9 and ascr#11 profile during** ***S. feltiae*** **development.** For each time point, six insect cadavers were analyzed by LC-MS. For the 0 time point, 4 uninfected larvae were analyzed. * detected but not quantifiable.
(TIF)

Figure S4 **Ascr#9 can replace the function of ascr#2 in the** ***C. elegans*** **dispersal blend.** Representative pictures are presented. Negative control using 0.25% *E. coli* HB101 (3 experiments), ascr#9 substitution in the *C. elegans* dispersal blend (6 experiments), and positive controls: the synthetic blend dispersal blend (7 experiments) and positive control dauer liquid culture (3 experiments).
(TIF)

Figure S5 **Dispersal blend of the phylogenetically related nematodes species.** (A) Phylogenetic tree for entomopathogenic nematodes, plant parasitic nematodes and *C. elegans*. The figure is adapted from *C. elegans* and the biology of nematodes [7]. Red color indicates the example of genera. (B) Host insect cadaver of *Steinernema* spp. and *Heterorhabditis* spp. For each species, four insect (*G. mellonella*) cadavers infected with both *Steinernema* spp. or *Heterorhabditis* spp. were analyzed by LC-MS for ascr#9 and ascr#11 profiles.
(TIF)
