# Supplementary figures and images for: Interspecific Nematode Signals Regulate Dispersal Behavior
Source: PLoS One. 2012 Jun 6;7(6):e38735. doi: 10.1371/journal.pone.0038735 (PMC3368880; doi:10.1371/journal.pone.0038735)

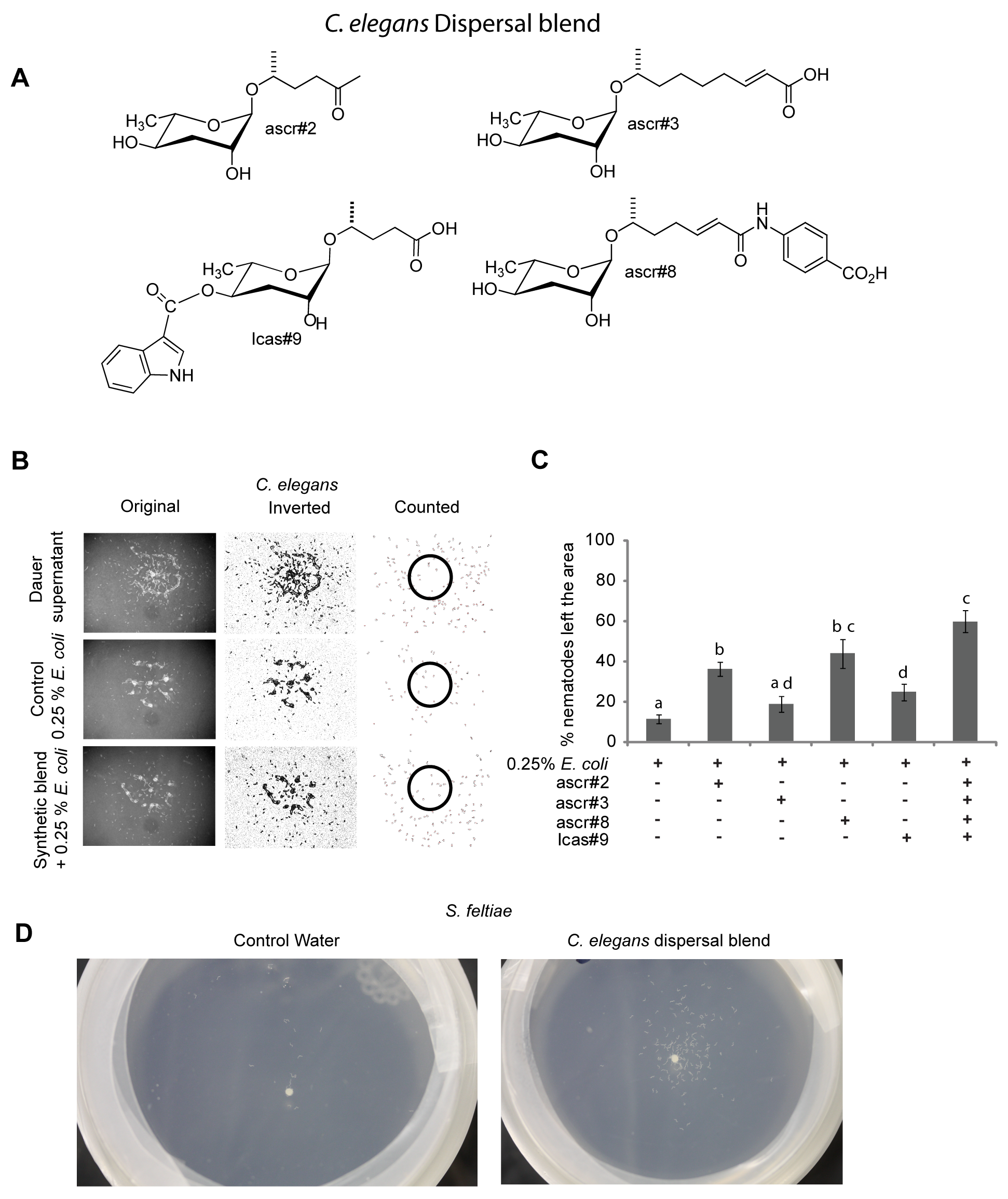

Supplement: Figure S1 — C. elegans dispersal blend, quantification and response of S. feltiae to C. elegans dispersal blend. (A), Structures of the ascarosides for the dispersal blend. (B), Quantification of dispersing C. elegans using Image J. Second column shows inverted pictures and the third column shows the counted nematodes. (C), Contribution of individual ascarosides to the activity of the synthetic blend. Ascr#2, (3.68 pmol/µl), ascr#3 (0.165 pmol/µl), ascr#8 (0.25 pmol/µl), and icas#9 (0.005 pmol/µl). Seven experiments were done for each treatment. +, present and −, absent. Student's t-test, unpaired (p<0.05). (D), S. feltiae response to C. elegans dispersal blend visualized within an entire plate. (TIF) [file pone.0038735.s004.tif]

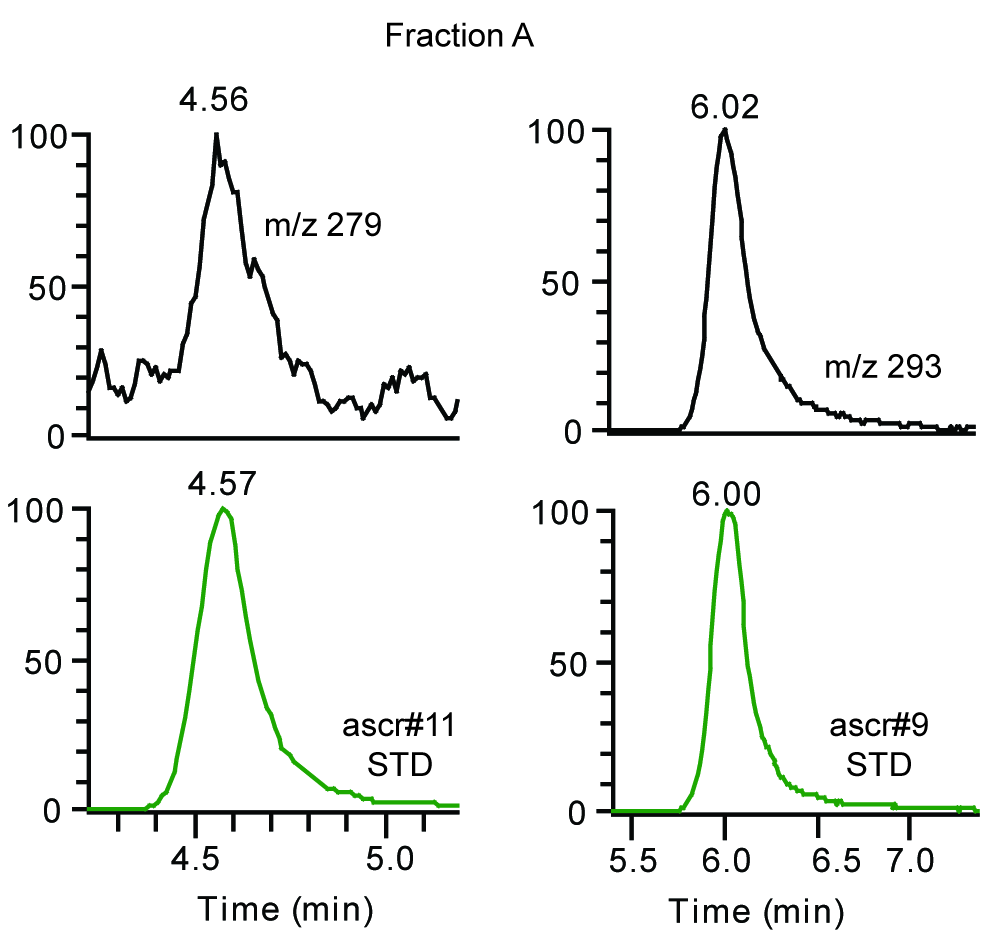

Supplement: Figure S2 — LC-MS ion chromatograms of fraction A. First panel shows ascr#11 at m/z 279, second panel shows ascr#9 at m/z 293. (TIF) [file pone.0038735.s005.tif]

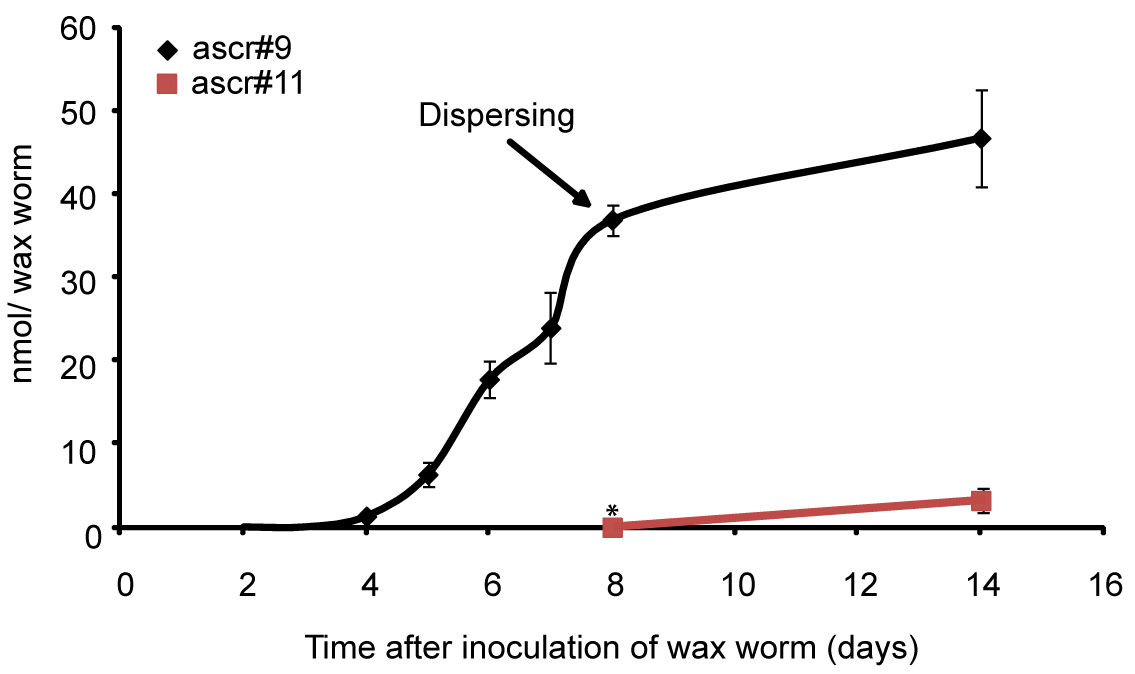

Supplement: Figure S3 — Ascr#9 and ascr#11 profile during S. feltiae development. For each time point, six insect cadavers were analyzed by LC-MS. For the 0 time point, 4 uninfected larvae were analyzed. * detected but not quantifiable. (TIF) [file pone.0038735.s006.tif]

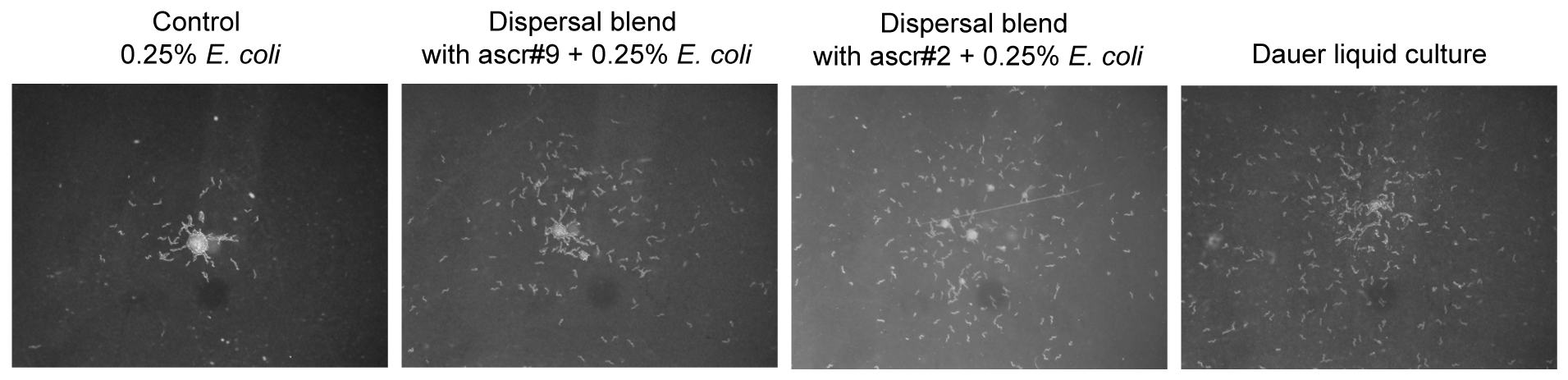

Supplement: Figure S4 — Ascr#9 can replace the function of ascr#2 in the C. elegans dispersal blend. Representative pictures are presented. Negative control using 0.25% E. coli HB101 (3 experiments), ascr#9 substitution in the C. elegans dispersal blend (6 experiments), and positive controls: the synthetic blend dispersal blend (7 experiments) and positive control dauer liquid culture (3 experiments). (TIF) [file pone.0038735.s007.tif]

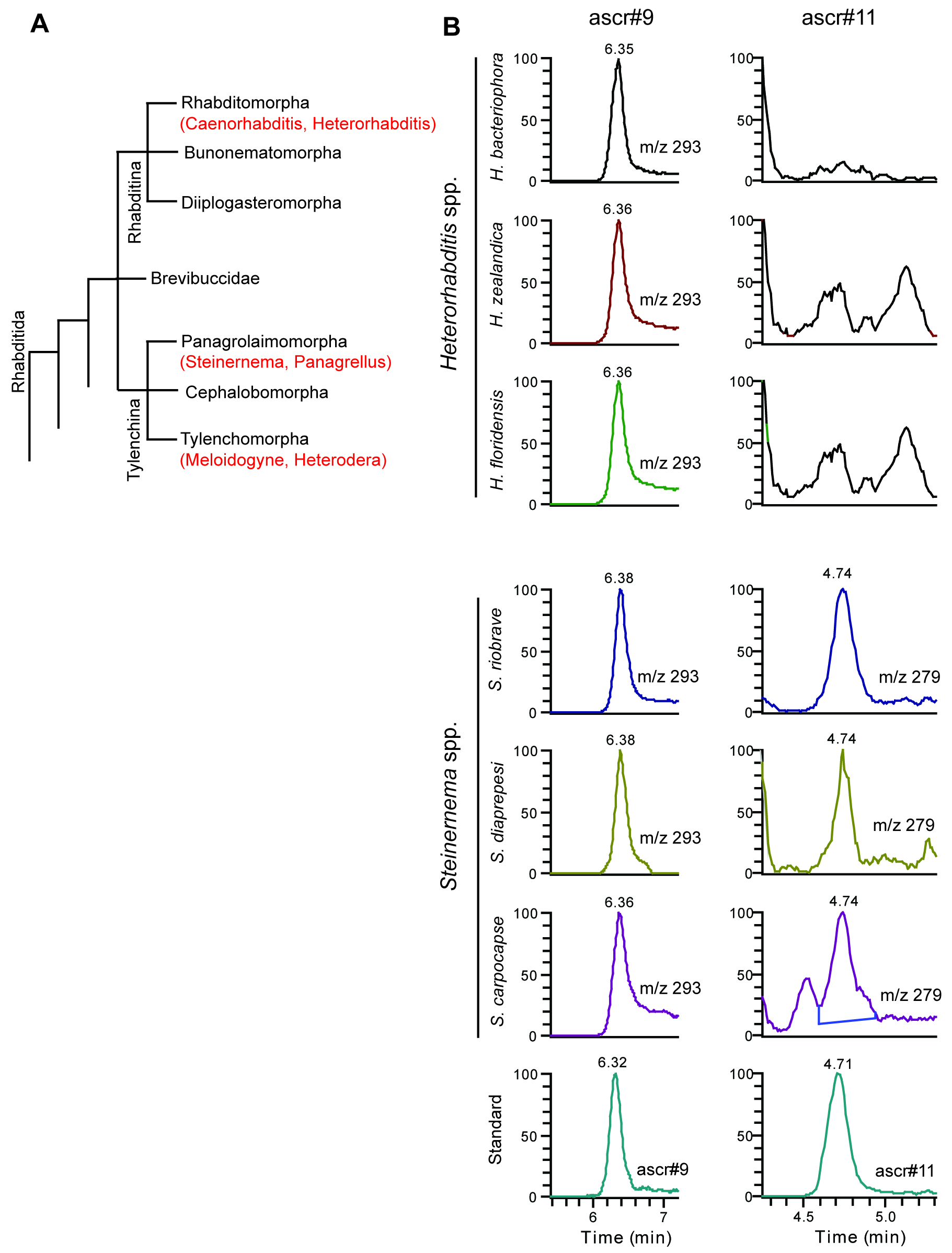

Supplement: Figure S5 — Dispersal blend of the phylogenetically related nematodes species. (A) Phylogenetic tree for entomopathogenic nematodes, plant parasitic nematodes and C. elegans. The figure is adapted from C. elegans and the biology of nematodes [7]. Red color indicates the example of genera. (B) Host insect cadaver of Steinernema spp. and Heterorhabditis spp. For each species, four insect (G. mellonella) cadavers infected with both Steinernema spp. or Heterorhabditis spp. were analyzed by LC-MS for ascr#9 and ascr#11 profiles. (TIF) [file pone.0038735.s008.tif]
